# SkipCPP-Pred: an improved and promising sequence-based predictor for predicting cell-penetrating peptides

**DOI:** 10.1186/s12864-017-4128-1

**Published:** 2017-10-16

**Authors:** Leyi Wei, Jijun Tang, Quan Zou

**Affiliations:** 1 School of Computer Science and Technology, Tianjin University, Tianjin, 30050 China; 2 State Key Laboratory of Medicinal Chemical Biology, Nankai University, Tianjin, 300074 China

**Keywords:** Cell-penetrating peptide, Machine learning, Adaptive k-skip-n-gram features

## Abstract

**Background:**

Cell-penetrating peptides (CPPs) are short peptides (5–30 amino acids) that can enter almost any cell without significant damage. On account of their high delivery efficiency, CPPs are promising candidates for gene therapy and cancer treatment. Accordingly, techniques that correctly predict CPPs are anticipated to accelerate CPP applications in future therapeutics. Recently, computational methods have been reportedly successful in predicting CPPs. Unfortunately, the predictive performance of existing methods is not satisfactory and reliable so as to accurately identify CPPs.

**Results:**

In this study, we propose a novel computational predictor called SkipCPP-Pred to further improve the predictive performance. The novelty of the proposed predictor is that we present a sequence-based feature representation algorithm called adaptive k-skip-n-gram that sufficiently captures the intrinsic correlation information of residues. By fusing the proposed adaptive skip features with a random forest (RF) classifier, we successfully construct the prediction model of SkipCPP-Pred. The various jackknife results demonstrate that the proposed SkipCPP-Pred is 3.6% higher than state-of-the-art CPP predictors in terms of accuracy. Moreover, we construct a high-quality benchmark dataset by reducing the data redundancy and enhancing the similarity between the positive and negative classes. Using this dataset to build prediction models, we can successfully avoid the performance bias lying in existing methods and yield a promising predictive model.

**Conclusions:**

The proposed SkipCPP-Pred is a simple and fast sequence-based predictor featured with the adaptive k-skip-n-gram model for the improved prediction of CPPs. Currently, SkipCPP-Pred is publicly available from an online webserver (http://server.malab.cn/SkipCPP-Pred/Index.html).

**Electronic supplementary material:**

The online version of this article (10.1186/s12864-017-4128-1) contains supplementary material, which is available to authorized users.

## Background

Cell-penetrating peptides (CPPs) are short peptides usually comprising 5–30 amino acid residues. Also known as protein transduction domains (PTDs), membrane translocating sequences (MTSs), and Trojan peptides, CPPs can directly enter cells without significantly damaging the cell membrane [1–3]. This unique ability of CPPs could be exploited to improve the cellular uptake of various bioactive molecules, which is inherently poor because bioactive cargoes tend to become trapped in the endosomes. When transported by CPPs, these cargoes are immediately freed in the cytosol to reach their intracellular targets (immediate bioavailability). CPPs are considered as very promising tools for non-invasive cellular import of cargoes, and have been successfully applied in in vitro and in vivo delivery of therapeutic molecules (e.g., small chemical molecules, nucleic acids, proteins, peptides, liposomes and particles). They also offer great potential as future therapeutics [3, 4] such as gene therapy and cancer treatments. The medical applicability of CPPs would be further enhanced by correct classification of peptides into CPPs or non-CPPs.

The first CPP, namely the Tat peptide, was derived from the transcription activator of human immunodeficiency virus type 1 in the late 1980s [5]. Since the discovery of Tat, hundreds of CPPs have been identified. The CPP-specific database CPPsite2.0 [6] currently contains 1850 experimentally validated CPPs, nearly double the contents of the previous version (CPPsite) [7]. As reported in [6], most of the known true CPPs are derived from natural proteins. The rapid development of next-generation sequencing techniques has revealed an increasing number of novel proteins, many of which might contain novel CPPs. However, predicting CPPs by traditional experimental methods is time-consuming and expensive. Thus, there is an urgent demand for fast prediction by computational methods. Most of the recent computational methods are based on machine-learning algorithms, which can automatically predict the cell-penetrating capability of a peptide. Although machine-learning-based methods have intrinsic advantages (time- and cost-saving) over experimental methods, they are less reliable than experimental methods. Therefore, they can play only a complementary role to experimental methods. Consequently, improving the predictive ability of computational predictors has been the major concern in this field.

Two factors [8], feature representation and classifier construction, are closely associated with the predictive performance of machine learning methods [9], and are largely responsible for the differences in existing de novo methods. For example, Sanders et al. [10] specified 61 representative physicochemical features of CPPs and built a prediction model using the support vector machine (SVM) classifier on a benchmark dataset including 111 known CPPs and 34 known non-CPPs. Their method achieved an overall accuracy of 75.86%. Exploiting the high efficiency of the SVM classifier, Gautam et al. [11] proposed a SVM-based predictor called CellPPD. They built multiple prediction models by considering various features such as amino acid and dipeptide compositions, binary pattern profiles, and physicochemical properties. In CellPPD, they also constructed a new larger benchmark dataset (784 true CPPs and an equal number of non-CPPs) that alleviates a major limitation of previous methods, namely, the small size of the training dataset (<111). To improve the robustness of prediction models, Holton et al. [12] proposed CPPpred, which trains the prediction model using an N-to-1 neural network. The training reduces the redundancy of the dataset by removing the 80% sequence similarity, which notably improves the prediction accuracy. More recently, Chen et al. [13] constructed a random forest (RF) prediction model that incorporates the well-known PseAAC (Pseudo Amino Acid Composition) with physicochemical properties developed by Chou [14]. They reported that a well-established feature selection algorithm improves the predictive performance.

As mentioned above, it is argued that the high predictive performance of existing computational methods seems doubtful [15–21]. First, the benchmark datasets used in the literature are too small to yield statistical results. For example, each of the four datasets constructed by Sanders et al. [10] contains fewer than 111 true CPPs. Besides being statistically insufficient, existing benchmark datasets are highly redundant, which biases the prediction results. For instance, the sequences in the current largest dataset proposed by Gautam et al. [11] share high sequence similarity. However, the high performance of their method on their proposed dataset (>90% accuracy) is probably not generalizable to other datasets. Therefore, a representative benchmark dataset is essential for robust CPP prediction by computational methods.

In this study, we propose a high-quality dataset for predicting CPPs. The high-quality of the dataset lies in three aspects. First, the dataset shares relatively low sequence similarity, with no more than 80%, avoid the bias in the performance. Second, the new CPP dataset is sufficiently large to build prediction models. Third, the dataset considers the importance of negative samples on predictive performance [22]. The collected negative samples (non-CPPs) are strictly based on the distribution of true CPPs in the dataset. To the best of our knowledge, the proposed dataset is the most stringent benchmark dataset in the literature. Using this dataset, we then train a novel CPP prediction method called SkipCPP-Pred, where we present an adaptive k-skip feature representation algorithm that sufficiently captures the correlation information of residues and successfully build the prediction model based on the RF classifier. As demonstrated by jackknife results on the proposed new dataset, the accuracy (ACC) of SkipCPP-Pred is 3.6% higher than that of state-of-the-art methods. The proposed SkipCPP-Pred is freely available from an online server (http://server.malab.cn/SkipCPP-Pred/Index.html ), and is anticipated to become an efficient tool for researchers working with CPPs.

## Methods

### Framework of the proposed method

**Fig. 1:**
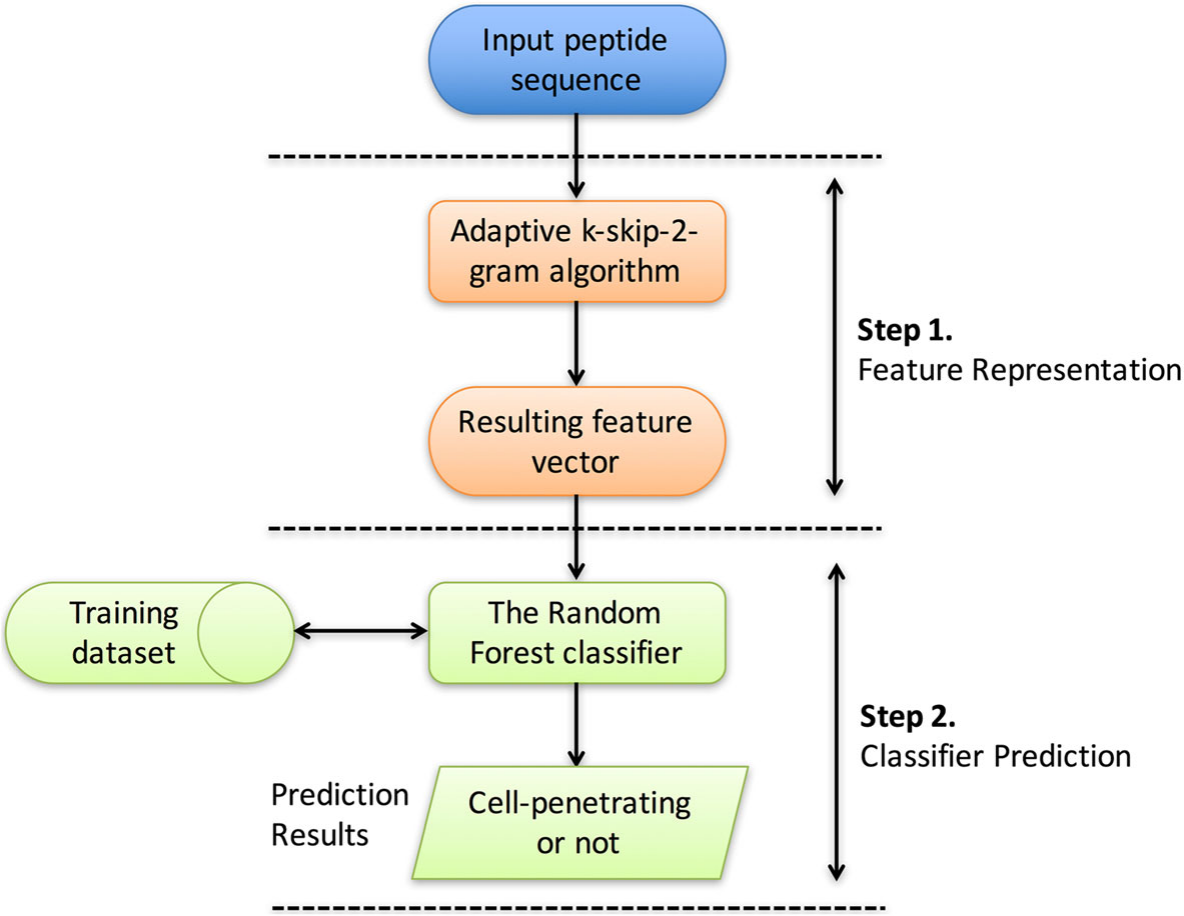
Framework of the proposed cell-penetrating peptide method SkipCPP-Pred

Figure 1 illustrates the framework of the proposed CPP prediction method SkipCPP-Pred. Below, we briefly describe the prediction process of a given peptide sequence or amino acid sequence in SkipCPP-Pred. In the first step, the sequence is submitted to the feature representation scheme, in which the proposed adaptive k-skip-2-g feature algorithm formulates the sequence into a fixed-length encoding (a 400-dimensional (400-D) feature vector). In the second step, the resulting feature vector is fed into a model trained by the underlying RF classifier, which predicts the cell-penetrating capability of the query peptide sequence. The proposed feature representation methods and the underlying classifier are detailed in the following sections.

### Feature representation

The k-skip-n-gram model, pioneered by Guthrie et al. [23], integrates the distance information into the traditional n-gram model [24]. To clarify the concept of the k-skip-n-gram model, we first introduce the traditional n-gram model.

For convenience, we denote a given amino acid sequence *S* as *A*_*1*_ *A*_*2*_ *A*_*3*_ *…A*_*L-1*_ *A*_*L*_, where *L* represents the length of the sequence; and the indices denote the positions of the amino acids in the sequence (for example, *A*_*1*_ and *A*_2_ are the first and second amino acids in *S*, respectively). *A*_*i*_ (1 ≤ *i* ≤ *L*) belongs to a set of 20 different amino acids, alphabetically ordered as Ω = {*A*, *C*, *D*, *E*, *F*, *G*, *H*, *I*, *K*, *L*, *M*, *N*, *P*, *Q*, *R*, *S*, *V*, *W*, *Y*}. An element of Ω is denoted as ${\Omega}_{i^{\prime }}$, where 1 ≤ *i* ^′^ ≤ 20; for example, Ω_1_ and Ω_2_ denote the first and seconds elements of Ω, respectively.

The traditional n-gram model provides the composition of n contiguous residues (*A*_*i*_ *A*_*i + 1*_ *…A*_*i + n-1*_) in the sequence *S*. To transform the variable sequence length into fixed length feature vectors, the traditional n-gram features are computed as follows:

1 $$ {FV}_{Grams}=\left\{\frac{N\left({\Omega}_{m_1}{\Omega}_{m_2}\dots {\Omega}_{m_n}\right)}{N\left({T}_{Grams}\right)}\right|1\le {m}_1\le 20,\kern0.5em 1\le {m}_2\le 20,\dots, 1\le {\Omega}_{m_n}\le 20\Big\}, $$

where *T*_*grams*_ = {*A*_*i*_ *A*_*i* + 1_ … *A*_*i* + *n* − 1_| 1 ≤ *i* ≤ *L* − *n* + 1} represents the set of segments with *n* spatially consecutive amino acids in *S*, and *N*(*T*_*Grams*_) denotes the total number of all elements in the set *T*_*grams*_. ${\Omega}_{m_1}{\Omega}_{m_2}\dots {\Omega}_{m_n}$are the 20^n^ possible residue combinations with length *n*. $N\left({\Omega}_{m_1}{\Omega}_{m_2}\dots {\Omega}_{m_n}\right)$ denotes the total number of the terms ${\Omega}_{m_1}{\Omega}_{m_2}\dots {\Omega}_{m_n}$ appearing in *T*_*grams*_. Accordingly, *FV*_*Grams*_ measures the occurrence frequencies of ${\Omega}_{m_1}{\Omega}_{m_2}\dots {\Omega}_{m_n}$ in *S*. The dimension of *FV*_*Grams*_ is 20^n^.

Clearly, the traditional n-gram model is sparse when the sequence *S* is short. To address this problem, the k-skip-n-gram model integrates the distance information into the n-gram model. The distance between any two residues *A*_*i*_ and *A*_*j*_ in a given sequence *S* is given by the interval length between the residues, calculated as

2 $$ DT\left({A}_i,{A}_j\right)=j-i-1. $$

For example, if *A*_*1*_ and *A*_*2*_ are contiguous, they are separated by an interval of zero length (i.e., no interval), and DT(*A*_*1,*_ *A*_*2*_) = 0, *A*_*1*_ and *A*_*3*_ are separated by one residue (*A*_*2*_), so the interval length DT(*A*_*1,*_ *A*_*3*_) = 1. Similarly, DT(*A*_*1,*_ *A*_*4*_) = 2 indicates that *A*_*1*_ and *A*_*4*_ are separated by two residues (*A*_*2*_ and *A*_*3*_).

The k-skip-n-gram model provides the composition of *n* residues with distances ≤k in *S*. In other words, in addition to the *n* contiguous residues considered in the traditional n-gram model, this model considers the *n* residues with distances 1 to *k* in S. Similar to the n-gram features, the k-skip-n-gram features are calculated as

3 $$ {FV}_{SkipGram}=\left\{\frac{N^{\prime}\left({\varOmega}_{m_1}{\varOmega}_{m_2}\dots {\varOmega}_{m_n}\right)}{N\left({T}_{SkipGram}\right)}\right|\ 1\le {m}_1\le 20,1\le {m}_2\le 20,\dots, 1\le {\Omega}_{m_n}\le 20\Big\}, $$

where *N*(*T*_*SkipGram*_) denotes the total number of all elements in the set *T*_*SkipGram*_, and ${N}^{\prime}\left({\Omega}_{m_1}{\Omega}_{m_2}\dots {\Omega}_{m_n}\right)$ denotes the total number of the terms ${\Omega}_{m_1}{\Omega}_{m_2}\dots {\Omega}_{m_n}$ appearing in the set *T*_*SkipGram*_, which is formulated as

4 $$ {T}_{SkipGram}=\left\{{\bigcup}_{a=1}^k Skip\left( DT=a\right)\right\} $$

where *Skip*(*DT* = *a*) = {*A*_*i*_ *A*_*i* + *a* + 1_ … *A*_*i* + *a* + *n* − 1_| 1 ≤ *i* ≤ *L* − *a* ,  1 ≤ *a* ≤ *k*}.

**Fig. 2:**
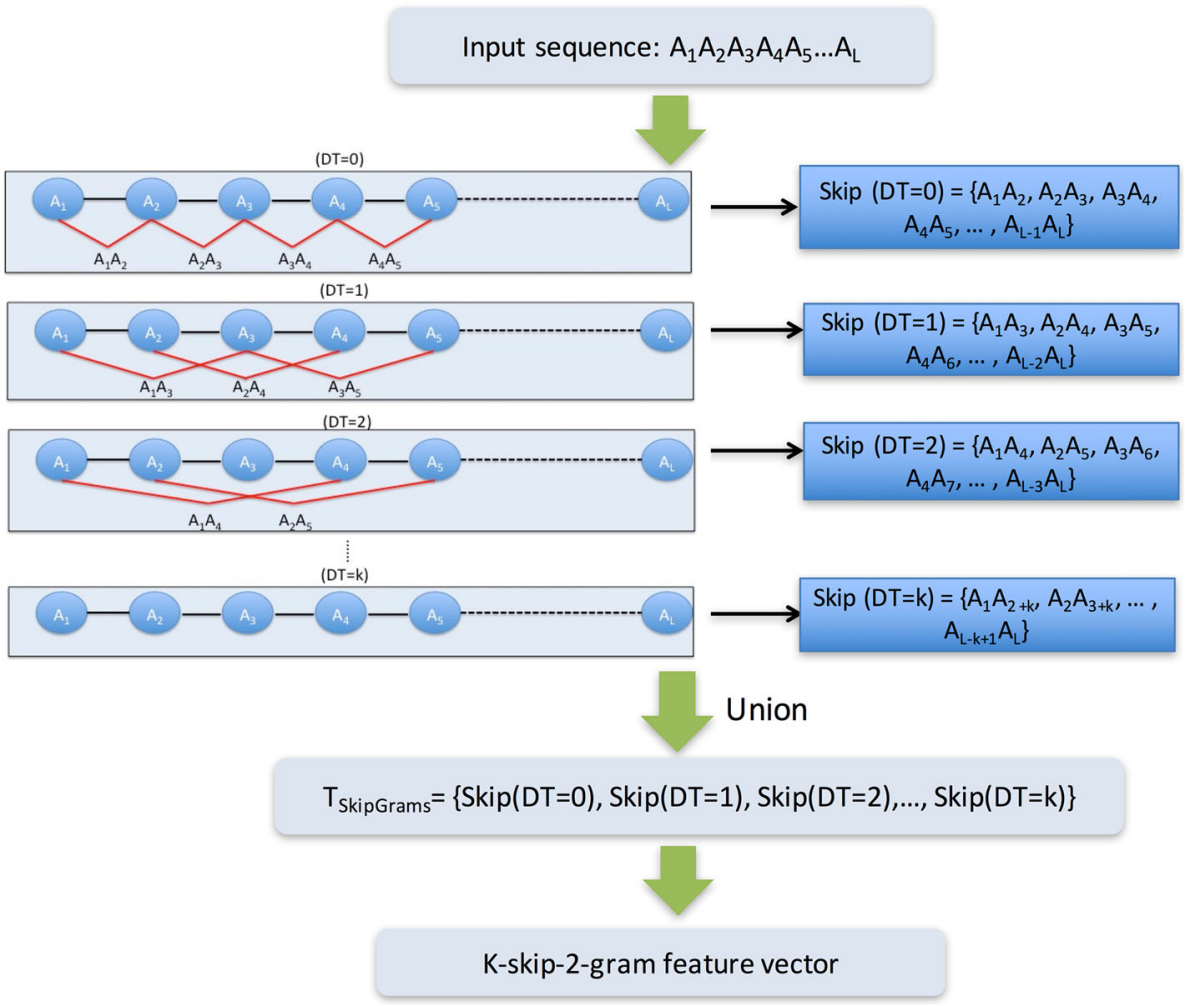
Schematic of the extraction scheme for k-skip-2-g features

Because the dimensions of the feature space exponentially expand with *n*, leading to the overfitting problem, we limit our analysis to *n*<3 (<20^3^-D). Moreover, when *n* = 1, the k-skip-n-gram model reduces to the traditional n-gram model. Thus, we analyze only the case for *n* = 2. The feature extraction procedure of the k-skip-2-g model is depicted in Fig. 2.

By definition of the k-skip-n-gram model, the parameter *k* measures the distance between any two residues. The maximum value of *k* should be the minimum sequence length in the dataset. However, almost all CPP sequences are short (5–30 residues), implying that *k* should not exceed 5. In other words, the skip features consider only the local distance information with the interval length no more than 5 in all sequences in the dataset, which would not properly reflect the varying distances in the dataset. Therefore, we proposed a modified strategy in which *k* is the length of each sequence during the skip feature extraction. In this way, the proposed feature representation algorithm becomes parameter free. Additionally, the feature algorithm adapts to the different lengths of sequences in the dataset, and includes more distance information in the features. The k-skip-n-gram features extracted by this new strategy are referred to as adaptive k-skip-n-gram features.

### Underlying classifier-Random Forest

The RF classifier, introduced by Breiman et al. [25], has proven to be a powerful classification algorithm in multiple Bioinformatics fields [26–28]. It constitutes an ensemble of decision trees (base classifiers) combined with a powerful ensemble strategy called modified bagging [25]. In this sense, the RF classifier behaves somewhat like an ensemble classifier [29, 30]. Unlike the traditional bagging algorithm, which uses all features to train each classifier, RF randomly selects a subset of features by a random feature selection technique, and grows a tree from those features (trains a base classifier). The required number of features for each base classifier is determined by computing the generalization error, classifier strength and dependence. The modified bagging algorithm enhances the diversity of the base classifiers, improving the efficiency of the traditional bagging algorithm. In our proposed method, the RF classifier is employed as the underlying classifier and is implemented in a data mining tool called WEKA (Waikato Environment for Knowledge Analysis) [31], an ensemble package of several machine learning algorithms. All experiments in this paper were carried out in WEKA 3.7.

### Dataset construction

According to machine learning theory, a well-established dataset is essential for building a robust and reliable prediction model. In this study, we carefully constructed an updated high-quality stringent dataset. The construction process of our dataset is described below.

#### Positive dataset construction

A CPP prediction dataset includes positive members (experimentally validated CPPs) and negative members (non-CPPs). The initial positive dataset was constructed from 1855 experimentally validated CPPs downloaded from the CPPsite2.0 database. Among these are 1564 natural and 291 non-natural CPPs. Natural CPPs are natural amino acid sequences, whereas in non-natural CPPs, some of the amino acids are replaced by artificial characters. As these non-natural CPPs cannot be formulated into fixed length feature vectors, they are excluded from the positive dataset. Redundancy in the dataset is known to bias the predictive performance of a model. Therefore, to improve the quality of the positive dataset, we removed the redundancies using the CD-HIT program [32], which has been widely applied in several fields [33–39]. Here, we set the similarity threshold in CD-HIT to 0.8, indicating that after reducing the sequence similarity, any two sequences in the positive differed by more than 80%. The elimination process retained 462 CPPs in the positive dataset.

#### Negative dataset construction

**Fig. 3:**
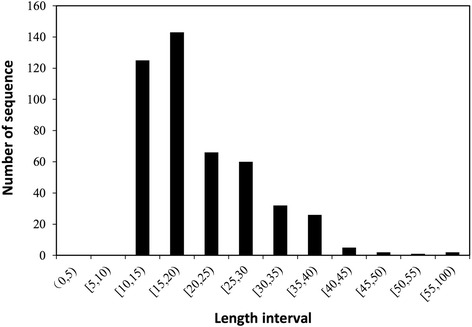
Distribution of true CPPs in the positive set. The *x*-axis denotes the length interval of the CPPs. For example, the interval (0, 5) denotes that the CPP is 1–4 amino acids long (greater than 0 and less than 5), whereas [5, 10] indicates a CPP length from 5 to 9 (greater than or equal to 5 and less than 10). The y-axis denotes the number of sequences (CPPs)

To construct the negative dataset, we randomly generated 2452 amino acid sequences with lengths between 5 and 50 as our initial negative pool. Such random generation of negative samples has been adopted in previous studies [10, 11]. For a high-quality and representative dataset, the number and distribution of the negative dataset must balance those of the positive dataset. To improve the similarity between the two datasets, the length distribution of the selected non-CPPs must match that of the positive dataset (see Fig. 3). To balance the data, we collected 462 non-CPPs into the negative dataset.

Ultimately, we successfully constructed a high-quality dataset containing 462 CPPs (positive samples) and 462 non-CPPs (negative samples). For convenience, our dataset is designated as CPP924. To our knowledge, CPP924 has the lowest data redundancy and the highest between-class similarity among the reported datasets. By virtue of the latter quality, our dataset is highly representative of real CPPs.

### Performance evaluation

The quality of the predictor was evaluated by evaluation metrics and a validation method.

Here, we employed four metrics commonly used in binary predictor evaluations [40]; sensitivity (SE), specificity (SP), accuracy (ACC), and Mathew’s correlation coefficient (MCC) [41]. These metrics are respectively formulated as

$$ \mathrm{SE}={\frac{TP}{TP+ FN}}^{\ast }100\% $$

$$ \mathrm{SP}={\frac{TN}{TN+ FP}}^{\ast }100\% $$

$$ \mathrm{ACC}={\frac{TP+ TN}{TP+ TN+ FN+ FP}}^{\ast }100\% $$

$$ \mathrm{MCC}={\frac{TP^{\ast } TN-{FP}^{\ast } FN}{\sqrt{\left( TP+ FN\right)\left( TP+ FP\right)\left( TN+ FP\right)\left( TN+ FN\right)}}}^{\ast }100\%, $$

where *TP*, *TN*, *FP* and *FN* represent the numbers of true positives, true negatives, false positives and false negatives, respectively. The first two metrics, SE and SP, measure the ability of the predictor to predict the correct class [42]. Specifically, the SE and SP compute the accuracy of predicting samples in the positive and negative classes, respectively. The other two metrics, ACC and MCC, comprehensively measure the predictive performance of a predictor. Computed on balanced data, higher ACC and MCC scores both imply a higher quality predictor. Computed on unbalanced data, the MCC more accurately reflects the predictive quality of a predictor than ACC.

The effectiveness of a prediction model must be tested by a validation method. The three statistical validation methods are *k*-fold cross validation, the jackknife test [28], and an independent test. Among these methods, the jackknife test is considered to best determine whether the method yields a unique result for a given benchmark dataset [43–54]. The jackknife test isolates each protein one by one and trains the predictor by the remaining proteins in the learning dataset. The jackknife test has been widely employed in performance validations of diverse predictors. Thus, it is adopted as the underlying validation of the proposed method.

### Guideline of webserver

An available webserver is important for researchers to access the proposed method to make predictions. Here, we have built a user-friendly webserver that implements the proposed method SkipCPP-Pred. The webserver is now freely accessible to the public. In this section, we give researchers a step-by-step guideline on how to use the webserver to get the predicted results they desire. The guideline is described as follows,

- **Step 1.** Go to the website (http://server.malab.cn/SkipCPP-Pred/Index.html) to see the homepage of the webserver. Click on the button **About** and you will see a brief introduction about how the proposed method SkipCPP-Pred is set up.
- **Step 2.** Enter the query protein sequences into the input box. The input sequences should be in the FASTA format. Examples of FASTA-formatted protein sequences can be seen by clicking on the button **FASTA format** above the input box. In particular, the webserver can receive an un-limited number of query sequences for every single run.
- **Step 3.** By clicking on the button **Predict**, you will get the predicted results on the screen of your computer. Take an actual cell-penetrating peptide, with an identifier of “cpp_P7–4”, as an example. After you enter the query sequence into the input box and click on the button **Predict**, you will see the predicted result showed on the screen: “Cell-penetrating” with the prediction confidence of 98.8%.
- **Step 4.** Click on the button **Clear** to delete the query sequences you enter to the input box.
- **Step 5.** Click on the button **Datasets** to download the benchmark datasets used in this paper and the feature sets we proposed based on the datasets.

## Results

### Feature comparison and contribution analysis

This study proposes adaptive k-skip-2-g features as modifications of the traditional 2-g features. To investigate the impact of the adaptive k-skip-2-g features, we compared their performances with those of the traditional 2-g features. Classifier bias was avoided by employing two high-efficiency classifiers (RF and LibSVM). Table 1 presents the jackknife results of both feature sets on the CPP924 dataset. For both classifiers, the adaptive k-gram-2-g features perform significantly better than the traditional 2-g features. In the RF results (row 3 of Table 1), the ACC of the adaptive k-gram-2-g features is 90.6%, 3.2% higher than that of the traditional 2-g features (87.4%). In the LibSVM results (row 4 of Table 1), the accuracy improvement of the adaptive k-gram-2-g features is 3.4%. This demonstrates that the discriminative information, by which true CPPs are distinguished from non-CPPs, is higher in the proposed adaptive k-skip-2-g features than in the traditional 2-g features. We infer that the extra discriminative power is conferred solely by the distance information of the amino acids in the sequence, because the two feature sets differ only by the additional distance information in the feature representation.

**Table 1:** Jackknife results of the adaptive k-skip-2-g features and traditional 2-g features evaluated on the CPP924 dataset

Classifiers	Adaptive k-skip-2-g features				Traditional 2-g features			
	SE (%)	SP (%)	ACC (%)	MCC	SE (%)	SP (%)	ACC (%)	MCC
RF	88.5	92.6	90.6	0.812	89.0	85.9	87.4	0.751
LibSVM	88.1	92.6	90.4	0.810	83.3	90.7	87.0	0.745

**Fig. 4:**
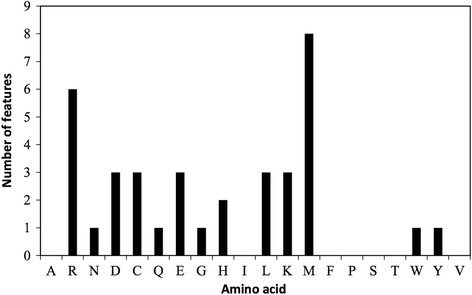
Amino acid compositions of the 20 most important features among 400 adaptive k-skip-2-g features. For example, *R* = 6 indicates that six out of the 20 top-scoring features contain the amino acid R; conversely, A = 0 indicates that none of the top-scoring features contain the amino acid A

We also analyzed the importance of the proposed k-skip-2-g features by calculating the information gain score [55]. This measure, denoted as IG(x, c), represents the information gain of feature x relative to the class attribute c. The higher the information gain score, the greater the discriminative power of the feature. Table 2 lists the 20 most important features among the 400 k-skip-2-g features; the ranking list of all 400 features can be found in Additional file 1: Table S1. As shown in Table 2, the highest information gain score (0.252) is gained by “RR”, indicating that the amino acid “R” is extremely useful for classifying true CPPs and non-CPPs. The same result has been reported in previous studies [56]. The classification powers of the 20 amino acids in the top-scoring features are illustrated in Fig. 4. We observe that eight of the 20 features contain the amino acid “M”, versus six features containing “R”. This demonstrates that “M” is at least as important as “R” for classification purposes. This discriminative power analysis of specific features in the feature set is anticipated to assist researchers working with CPPs.

**Table 2:** The 20 most important features among the 400 proposed adaptive k-skip-2-g features

Rank	IG(x, c)^a^	Features
1	0.252	RR
2	0.12	KR
3	0.119	KK
4	0.115	LR
5	0.113	MM
6	0.107	RK
7	0.107	DM
8	0.105	YM
9	0.105	ME
10	0.104	EM
11	0.103	LL
12	0.093	HM
13	0.093	DQ
14	0.092	RL
15	0.091	MH
16	0.091	DW
17	0.089	CE
18	0.088	CN
19	0.087	CM
20	0.087	GR

IG(x, c)^a^ is the information gain of feature x relative to the class attribute c. The higher the IG(x, c), the more discriminative the feature

### Classifier parameter optimization

**Fig. 5:**
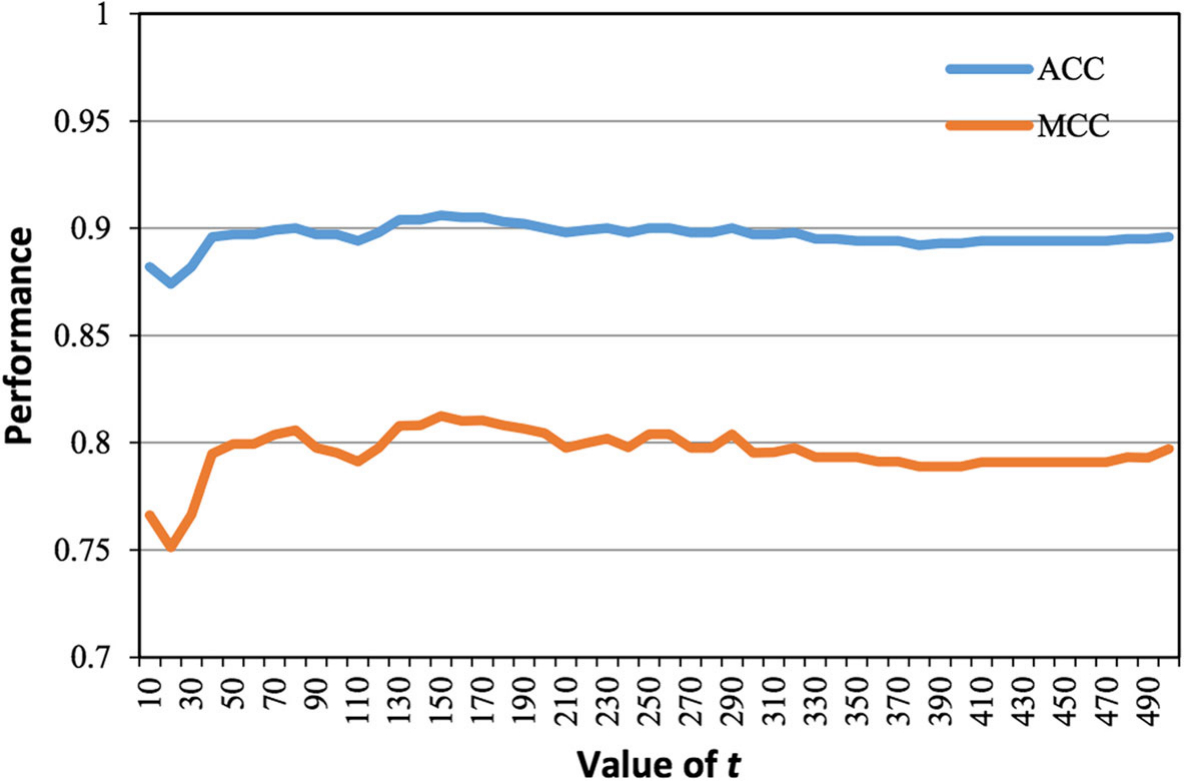
Performance of the RF classifier with different tree numbers. The x- and y-axes represent the tree number *t* (varied from 10 to 500 in steps of 10) and the predictive performance, respectively. The blue and orange plots present the comprehensive metrics ACC and MCC, respectively

Classifier parameter optimization is a potentially useful means of improving the predictive performance of machine learning methods [57–60]. Therefore, we also conducted an optimization experiment for the underlying RF classifier in the proposed method. The major parameter of the RF classifier is the tree number *t*, which can be any integer higher than 1. Here, we investigated the impact of varying *t* from 10 to 500 in 10-step increments, and executing the RF classifier on the CPP924 dataset. The jackknife results for the various *t* values are illustrated in Fig. 5. The RF exhibits its best performance at *t* = 150. Therefore, we set *t* = 150 in our prediction model. The prediction results of the RF classifier for different values of *t* are detailed in Additional file 1: Table S2.

### Performance of the underlying classifier

Table 3 lists the performance of the underlying RF classifier on the benchmark dataset CPP924, determined in the jackknife validation test. For comparison, the performance of five popular classifiers (LibSVM, Naïve Bayes, J48, SMO, and Logistic Regression) was evaluated on the same dataset. All of these classifiers were executed in WEKA 3.7. As shown in Table 3, the RF classifier achieved the best performance among the classifiers (with an ACC and MCC of 90.6% and 0.812, respectively). Note that RF performed similarly to LibSVM, but significantly outperformed the other classifiers (by 1.9%–9.2% and 0.039–0.184 in the ACC and MCC scores, respectively). These results consolidate RF and SVM as the best-performing (most efficient) classification algorithms in CPP determination. In conclusion, a well-trained RF classifier accurately discriminates true CPPs from non-CPPs.

**Table 3:** Jackknife results of the underlying random forest classifier and four alternative classifiers on the benchmark dataset CPP924

Classifier	SE (%)	SP (%)	ACC (%)	MCC
NB	82.7	94.8	88.7	0.781
SMO	87.9	89.4	88.6	0.773
J48	87.2	84.6	85.9	0.719
LR	82.0	80.7	81.4	0.628
LibSVM	88.1	92.6	90.4	0.810
RF	88.5	92.6	90.6	0.812

NB and LR denote Naïve Bayes and Logistic Regression, respectively

### Comparison with state-of-the-art predictors

To evaluate the effectiveness of the proposed computational predictor SkipCPP-Pred, we compared its performance with that of CellPPD, the best-performing predictor in the literature [11]. In this comparison, the CellPPD predictor alone was selected because this predictor is known to outperform other existing predictors [11]. Therefore, comparisons with other computational predictors are redundant here. As the proposed SkipCPP-Pred is a sequence-based predictor, it was tested against two sequenced-based predictive models of CellPPD: (1) dipeptide composition model and (2) binary profile-based model. For convenience of discussion, the two predictive models of CellPPD are denoted as CellPPD-DC and CellPPD-BP, respectively. For a fair comparison, both methods (models) were tested under their optimal parameters.

The prediction results of the proposed SkipCPP-Pred and CellPPD, executed on the proposed high-quality dataset and evaluated by the jackknife test, are presented in Table 4. The overall accuracies (ACC) of SkipCPP-Pred, CellPPD-DC, and CellPPD-BP are 90.6%, 87.0%, and 83.7%, respectively. Note that SkipCPP-Pred remarkably outperforms the CellPPD predictor, which is 3.6% and 6.9% more accurate than CellPPD-DC and CellPPD-BP, respectively. This indicates that our predictor is superior to the CellPPD predictor for classification of CPPs. Moreover, it is worth noting that although CellPPD is reported to achieve >90% accuracy in the prediction of CPPs, its performance significantly declined to around 83% - 87% on our CPP924 dataset. This may verify our assumption that our dataset is more stringent than the dataset proposed in the CellPPD study.

**Table 4:** Jackknife results of the proposed SkipCPP-Pred and the state-of-the-art predictor CellPPD on the CPP924 dataset

Methods	SE (%)	SP (%)	ACC (%)	MCC
CellPPD-DC	83.3	90.7	87.0	0.745
CellPPD-BP	78.1	89.2	83.7	0.680
SkipCPP-Pred	88.5	92.6	90.6	0.812

Note that CellPPD-DC represents the dipeptide composition model of the CellPPD predictor, while CellPPD-BP represents the binary profile-based model of the CellPPD predictor

**Fig. 6:**
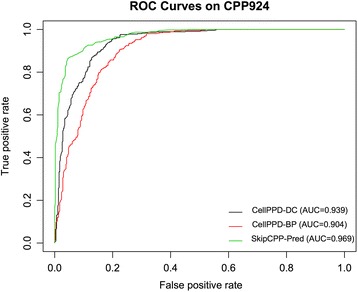
Graphical illustration to show the predictive performance of the proposed SkipCPP-Pred and the state-of-the-art predictors by using the ROC curves

To intuitively compare the proposed method with the CellPPD predictor, we further conducted a graphic analysis by using the Receiver Operating Characteristic (ROC) curves [61]. In the ROC analysis, area under the receiver operating characteristic curve (AUC) is the major metric to evaluate the predictive performance of a predictor. The greater is the AUC value, the better is the predictor. Figure 6 plots the ROC curves of the compared methods on the CPP924 dataset. As seen from Fig. 6 that the area under the curve of our method (green curve in Fig. 6) is significantly greater than that under the other curves (purple curve for CellPPD-DC and red curve for CellPPD-BP). To be specific, the AUC value of the proposed SkipCPP-Pred is 0.969, which is 0.03 and 0.065 higher than the CellPPD-DC and CellPPD-BP method, respectively. This further demonstrates that the proposed predictor is better than the state-of-the-art predictors.

## Discussion

To identify known and potential novel CPPs, we developed a predictor, namely SkipCPP-Pred, by using a sequence-based feature representation approach (adaptive k-skip-n-gram model).

To verify the effectiveness of the adaptive k-skip-n-gram model, we compared it with traditional n-gram model and found that the adaptive k-skip-n-gram model shows better performance than the traditional n-gram model. This is because that as compared with the tradition n-gram model, additional distanced correlation information embedded in the adaptive k-skip-n-gram model contributes to the performance improvement for CPP prediction.

Moreover, due to the use of only sequential information for modeling, SkipCPP-Pred is capable to predict CPPs fast. We tested SkipCPP-Pred on the stringent CPP924 dataset with the jackknife test. The results indicated that SkipCPP-Pred has the strong capacity of classifying true CPPs from non-CPPs. And, we developed an online webserver that implements the proposed SkipCPP-Pred for researchers to predict CPPs conveniently. It is anticipated to be a useful tool to accelerate the research of CPP prediction. Importantly, the proposed feature representation method used in SkipCPP-Pred has the great potential to guide the sequence-based prediction of other special proteins (i.e. DNA-binding proteins).

## Conclusions

In this study, we proposed a novel computational method called SkipCPP-Pred, for accurate, fast and stable prediction of potential novel CPPs. Recognizing the importance of the dataset in model building, we also proposed a novel high-quality dataset for SkipCPP-Pred. The quality of this dataset is guaranteed by reducing the sequence redundancy, which alleviates the bias in the performance, and enhancing the similarity between the two classes (positive and negative CPPs). To our knowledge, we present the most stringent of the datasets reported in the literature. Thus, our high-quality dataset might become the benchmark dataset in the development of computational CPP prediction methods. As another contribution, we proposed the adaptive k-skip-n-gram model to CPP prediction. By the feature comparative analysis, the k-skip-n-gram feature model demonstrated greater discriminative power in CPP classification than the traditional n-gram model. Moreover, we compared the overall performance of the proposed SkipCPP-Pred and the state-of-the-art predictors in the literature. The jackknife results showed that the ACC and MCC performance measures on the CPP924 dataset were higher in SkipCPP-Pred than in the state-of-the-art predictors, demonstrating the superiority of SkipCPP-Pred. Accordingly, it is expected that the proposed predictor could become a useful tool in research of CPP prediction. At least, it could complement the existing predictors to improve the accuracy of CPP prediction by neural-like computing models [62–67], evolutionary computation [68, 69], and other similar models [70–73] in near future.

## Additional file

Additional file 1: Table S1. Feature ranking of the proposed adaptive k-skip-2-g features. IG(x,c)^a^ denotes information gain score. Higher IG(x,c) for a feature means the feature is more discriminative. **Table S2.** Performance of the Random Forest classifier with different tree numbers on the benchmark dataset CPP924 with the jackknife validation test. Note that the tree number is changed from 10 to 500 with the incremental step of 10. (DOCX 61 kb)
