# Supplementary material for: SkipCPP-Pred: an improved and promising sequence-based predictor for predicting cell-penetrating peptides
Source: BMC Genomics. 2017 Oct 16;18(Suppl 7):742. doi: 10.1186/s12864-017-4128-1 (PMC5657092; doi:10.1186/s12864-017-4128-1)
Supplement: Supplementary file 1 — Feature ranking of the proposed adaptive k-skip-2-g features. IG(x,c)a denotes information gain score. Higher IG(x,c) for a feature means the feature is more discriminative. Table S2. Performance of the Random Forest classifier with different tree numbers on the benchmark dataset CPP924 with the jackknife validation test. Note that the tree number is changed from 10 to 500 with the incremental step of 10. (DOCX 61 kb) [file 12864_2017_4128_MOESM1_ESM.docx]

**Additional file 1**

**Table S1.** **Feature ranking of the proposed adaptive k-skip-2-gram features.** IG(x,c)^a^ denotes information gain score. Higher IG(x,c) for a feature means the feature is more discriminative.

| Rank | IG(x,c)^a^ | Features |
| --- | --- | --- |
| 1 | 0.252 | RR |
| 2 | 0.12 | KR |
| 3 | 0.119 | KK |
| 4 | 0.115 | LR |
| 5 | 0.113 | MM |
| 6 | 0.107 | RK |
| 7 | 0.107 | DM |
| 8 | 0.105 | YM |
| 9 | 0.105 | ME |
| 10 | 0.104 | EM |
| 11 | 0.103 | LL |
| 12 | 0.093 | HM |
| 13 | 0.093 | DQ |
| 14 | 0.092 | RL |
| 15 | 0.091 | MH |
| 16 | 0.091 | DW |
| 17 | 0.089 | CE |
| 18 | 0.088 | CN |
| 19 | 0.087 | CM |
| 20 | 0.087 | GR |
| 21 | 0.086 | LK |
| 22 | 0.086 | HY |
| 23 | 0.084 | SH |
| 24 | 0.084 | YE |
| 25 | 0.083 | FH |
| 26 | 0.082 | HP |
| 27 | 0.082 | RS |
| 28 | 0.081 | VM |
| 29 | 0.08 | CY |
| 30 | 0.08 | HD |
| 31 | 0.078 | CD |
| 32 | 0.078 | DH |
| 33 | 0.078 | MD |
| 34 | 0.077 | MF |
| 35 | 0.077 | TM |
| 36 | 0.077 | CW |
| 37 | 0.076 | IH |
| 38 | 0.076 | DY |
| 39 | 0.076 | EF |
| 40 | 0.075 | HG |
| 41 | 0.075 | VH |
| 42 | 0.075 | AR |
| 43 | 0.074 | QE |
| 44 | 0.073 | DN |
| 45 | 0.072 | FM |
| 46 | 0.072 | HE |
| 47 | 0.072 | HR |
| 48 | 0.072 | DI |
| 49 | 0.071 | YW |
| 50 | 0.071 | QD |
| 51 | 0.07 | MY |
| 52 | 0.07 | HH |
| 53 | 0.07 | EW |
| 54 | 0.07 | KL |
| 55 | 0.07 | HV |
| 56 | 0.07 | YD |
| 57 | 0.069 | DE |
| 58 | 0.069 | EY |
| 59 | 0.069 | RP |
| 60 | 0.069 | HS |
| 61 | 0.069 | HT |
| 62 | 0.068 | HN |
| 63 | 0.068 | CG |
| 64 | 0.068 | FE |
| 65 | 0.068 | CQ |
| 66 | 0.067 | NE |
| 67 | 0.067 | CT |
| 68 | 0.067 | NH |
| 69 | 0.067 | MN |
| 70 | 0.066 | WH |
| 71 | 0.066 | WY |
| 72 | 0.066 | DC |
| 73 | 0.066 | MI |
| 74 | 0.066 | WE |
| 75 | 0.065 | WD |
| 76 | 0.065 | EH |
| 77 | 0.065 | CH |
| 78 | 0.065 | ND |
| 79 | 0.064 | MC |
| 80 | 0.064 | AL |
| 81 | 0.064 | HW |
| 82 | 0.064 | AM |
| 83 | 0.064 | PM |
| 84 | 0.064 | HI |
| 85 | 0.063 | ED |
| 86 | 0.063 | YY |
| 87 | 0.063 | NM |
| 88 | 0.063 | VE |
| 89 | 0.063 | YC |
| 90 | 0.063 | MT |
| 91 | 0.063 | TH |
| 92 | 0.062 | LH |
| 93 | 0.062 | VY |
| 94 | 0.062 | NY |
| 95 | 0.062 | SM |
| 96 | 0.062 | YN |
| 97 | 0.062 | IE |
| 98 | 0.061 | CC |
| 99 | 0.061 | VD |
| 100 | 0.06 | MW |
| 101 | 0.06 | VC |
| 102 | 0.06 | GK |
| 103 | 0.06 | NQ |
| 104 | 0.059 | VW |
| 105 | 0.059 | YH |
| 106 | 0.059 | NC |
| 107 | 0.059 | EV |
| 108 | 0.059 | EQ |
| 109 | 0.059 | NV |
| 110 | 0.058 | RA |
| 111 | 0.058 | CK |
| 112 | 0.058 | RH |
| 113 | 0.058 | WM |
| 114 | 0.057 | CV |
| 115 | 0.057 | CI |
| 116 | 0.057 | HF |
| 117 | 0.057 | YV |
| 118 | 0.057 | AK |
| 119 | 0.057 | EI |
| 120 | 0.057 | TE |
| 121 | 0.057 | AH |
| 122 | 0.056 | TC |
| 123 | 0.056 | EC |
| 124 | 0.056 | EN |
| 125 | 0.055 | HC |
| 126 | 0.055 | RG |
| 127 | 0.055 | IY |
| 128 | 0.055 | DD |
| 129 | 0.054 | PW |
| 130 | 0.054 | QY |
| 131 | 0.054 | CF |
| 132 | 0.054 | IM |
| 133 | 0.053 | VR |
| 134 | 0.053 | MV |
| 135 | 0.053 | MS |
| 136 | 0.053 | PE |
| 137 | 0.053 | SE |
| 138 | 0.053 | CR |
| 139 | 0.052 | GM |
| 140 | 0.052 | ID |
| 141 | 0.052 | HA |
| 142 | 0.052 | VN |
| 143 | 0.052 | WN |
| 144 | 0.052 | NW |
| 145 | 0.051 | CS |
| 146 | 0.051 | ML |
| 147 | 0.05 | AA |
| 148 | 0.05 | YS |
| 149 | 0.05 | HL |
| 150 | 0.05 | VQ |
| 151 | 0.05 | ET |
| 152 | 0.05 | GH |
| 153 | 0.05 | RC |
| 154 | 0.05 | YI |
| 155 | 0.049 | NN |
| 156 | 0.049 | EP |
| 157 | 0.049 | LA |
| 158 | 0.049 | TD |
| 159 | 0.048 | MQ |
| 160 | 0.048 | YA |
| 161 | 0.048 | QT |
| 162 | 0.048 | IC |
| 163 | 0.048 | HK |
| 164 | 0.048 | KP |
| 165 | 0.047 | HQ |
| 166 | 0.047 | AC |
| 167 | 0.047 | MG |
| 168 | 0.047 | IT |
| 169 | 0.046 | MP |
| 170 | 0.046 | MA |
| 171 | 0.046 | SW |
| 172 | 0.046 | EE |
| 173 | 0.046 | CL |
| 174 | 0.046 | YF |
| 175 | 0.046 | KA |
| 176 | 0.046 | DA |
| 177 | 0.046 | DK |
| 178 | 0.046 | QC |
| 179 | 0.046 | PH |
| 180 | 0.045 | DF |
| 181 | 0.045 | KY |
| 182 | 0.045 | PK |
| 183 | 0.045 | DV |
| 184 | 0.045 | FY |
| 185 | 0.045 | LD |
| 186 | 0.045 | SR |
| 187 | 0.044 | WW |
| 188 | 0.044 | PF |
| 189 | 0.044 | QM |
| 190 | 0.044 | SC |
| 191 | 0.044 | QH |
| 192 | 0.044 | IV |
| 193 | 0.044 | WT |
| 194 | 0.044 | FC |
| 195 | 0.044 | PC |
| 196 | 0.044 | WI |
| 197 | 0.044 | SY |
| 198 | 0.044 | SD |
| 199 | 0.043 | IR |
| 200 | 0.043 | DS |
| 201 | 0.043 | RW |
| 202 | 0.042 | FD |
| 203 | 0.042 | WC |
| 204 | 0.042 | CA |
| 205 | 0.042 | VF |
| 206 | 0.041 | IP |
| 207 | 0.041 | IW |
| 208 | 0.041 | YP |
| 209 | 0.041 | GE |
| 210 | 0.041 | RE |
| 211 | 0.041 | VT |
| 212 | 0.04 | LE |
| 213 | 0.04 | PI |
| 214 | 0.04 | DT |
| 215 | 0.04 | EK |
| 216 | 0.04 | AD |
| 217 | 0.039 | QW |
| 218 | 0.039 | RT |
| 219 | 0.039 | KH |
| 220 | 0.039 | VI |
| 221 | 0.038 | QV |
| 222 | 0.038 | RY |
| 223 | 0.038 | TY |
| 224 | 0.038 | LM |
| 225 | 0.038 | SK |
| 226 | 0.038 | DL |
| 227 | 0.038 | ES |
| 228 | 0.037 | WS |
| 229 | 0.037 | NS |
| 230 | 0.037 | RM |
| 231 | 0.037 | CP |
| 232 | 0.036 | TW |
| 233 | 0.036 | PD |
| 234 | 0.036 | VK |
| 235 | 0.036 | KE |
| 236 | 0.036 | QP |
| 237 | 0.035 | AY |
| 238 | 0.035 | KC |
| 239 | 0.035 | QI |
| 240 | 0.034 | LS |
| 241 | 0.034 | ST |
| 242 | 0.034 | GN |
| 243 | 0.034 | PQ |
| 244 | 0.034 | PN |
| 245 | 0.034 | WP |
| 246 | 0.034 | FF |
| 247 | 0.034 | DG |
| 248 | 0.034 | VS |
| 249 | 0.033 | KT |
| 250 | 0.033 | SN |
| 251 | 0.033 | YK |
| 252 | 0.033 | QF |
| 253 | 0.033 | NP |
| 254 | 0.032 | RD |
| 255 | 0.032 | QN |
| 256 | 0.032 | FN |
| 257 | 0.032 | TI |
| 258 | 0.032 | FT |
| 259 | 0.032 | KM |
| 260 | 0.032 | TN |
| 261 | 0.032 | YT |
| 262 | 0.031 | NF |
| 263 | 0.031 | PY |
| 264 | 0.031 | KD |
| 265 | 0.031 | MK |
| 266 | 0.031 | PT |
| 267 | 0.031 | FI |
| 268 | 0.031 | MR |
| 269 | 0.031 | AE |
| 270 | 0.031 | PR |
| 271 | 0.03 | QR |
| 272 | 0.03 | TQ |
| 273 | 0.03 | IS |
| 274 | 0.03 | PL |
| 275 | 0.03 | WQ |
| 276 | 0.029 | NT |
| 277 | 0.029 | FW |
| 278 | 0.029 | WV |
| 279 | 0.029 | PA |
| 280 | 0.029 | TF |
| 281 | 0.029 | VP |
| 282 | 0.029 | DR |
| 283 | 0.029 | NA |
| 284 | 0.028 | WR |
| 285 | 0.028 | NI |
| 286 | 0.028 | IL |
| 287 | 0.028 | FV |
| 288 | 0.028 | YQ |
| 289 | 0.028 | DP |
| 290 | 0.027 | LY |
| 291 | 0.027 | LP |
| 292 | 0.027 | QS |
| 293 | 0.026 | VG |
| 294 | 0.026 | AG |
| 295 | 0.026 | FQ |
| 296 | 0.026 | NL |
| 297 | 0.026 | GW |
| 298 | 0.026 | SF |
| 299 | 0.025 | GI |
| 300 | 0.025 | IQ |
| 301 | 0.025 | YL |
| 302 | 0.024 | WA |
| 303 | 0.024 | TL |
| 304 | 0.024 | EG |
| 305 | 0.024 | QG |
| 306 | 0.023 | RQ |
| 307 | 0.023 | IN |
| 308 | 0.023 | GD |
| 309 | 0.023 | GL |
| 310 | 0.023 | VA |
| 311 | 0.023 | EA |
| 312 | 0.023 | WG |
| 313 | 0.022 | FS |
| 314 | 0.022 | FA |
| 315 | 0.022 | LC |
| 316 | 0.022 | SI |
| 317 | 0.022 | TV |
| 318 | 0.021 | AF |
| 319 | 0.02 | AN |
| 320 | 0.02 | WF |
| 321 | 0.02 | GY |
| 322 | 0.02 | FP |
| 323 | 0.02 | GF |
| 324 | 0.019 | SP |
| 325 | 0.019 | SV |
| 326 | 0.019 | GG |
| 327 | 0.019 | RI |
| 328 | 0.018 | SS |
| 329 | 0.018 | YR |
| 330 | 0.018 | SQ |
| 331 | 0.018 | PP |
| 332 | 0.018 | FR |
| 333 | 0.017 | GQ |
| 334 | 0.016 | IK |
| 335 | 0.016 | IG |
| 336 | 0.015 | NG |
| 337 | 0.015 | LN |
| 338 | 0.015 | QA |
| 339 | 0.015 | AW |
| 340 | 0.015 | LI |
| 341 | 0.015 | AQ |
| 342 | 0.015 | GT |
| 343 | 0.015 | TR |
| 344 | 0.015 | QQ |
| 345 | 0.014 | GP |
| 346 | 0.014 | YG |
| 347 | 0.014 | VV |
| 348 | 0.014 | KV |
| 349 | 0.014 | KG |
| 350 | 0.014 | RV |
| 351 | 0.014 | AT |
| 352 | 0.013 | AI |
| 353 | 0.013 | NK |
| 354 | 0.013 | SG |
| 355 | 0.012 | EL |
| 356 | 0.012 | IF |
| 357 | 0.012 | LG |
| 358 | 0.011 | WK |
| 359 | 0 | KS |
| 360 | 0 | VL |
| 361 | 0 | KI |
| 362 | 0 | AP |
| 363 | 0 | AS |
| 364 | 0 | KW |
| 365 | 0 | GV |
| 366 | 0 | GS |
| 367 | 0 | KF |
| 368 | 0 | KQ |
| 369 | 0 | FG |
| 370 | 0 | LF |
| 371 | 0 | LQ |
| 372 | 0 | IA |
| 373 | 0 | LT |
| 374 | 0 | LW |
| 375 | 0 | II |
| 376 | 0 | LV |
| 377 | 0 | KN |
| 378 | 0 | AV |
| 379 | 0 | FL |
| 380 | 0 | NR |
| 381 | 0 | TG |
| 382 | 0 | TA |
| 383 | 0 | SL |
| 384 | 0 | TK |
| 385 | 0 | TP |
| 386 | 0 | TS |
| 387 | 0 | TT |
| 388 | 0 | WL |
| 389 | 0 | RF |
| 390 | 0 | PV |
| 391 | 0 | RN |
| 392 | 0 | FK |
| 393 | 0 | GC |
| 394 | 0 | QL |
| 395 | 0 | GA |
| 396 | 0 | PG |
| 397 | 0 | ER |
| 398 | 0 | PS |
| 399 | 0 | QK |
| 400 | 0 | SA |

**Table S2. Performance of the Random Forest classifier with different tree numbers on the benchmark dataset CPP924 with the jackknife validation test.** Note that the tree number is changed from 10 to 500 with the incremental step of 10.

| Tree Number (*t*) | SE | SP | ACC | MCC |
| --- | --- | --- | --- | --- |
| 10 | 0.892 | 0.872 | 0.882 | 0.766 |
| 20 | 0.864 | 0.885 | 0.874 | 0.751 |
| 30 | 0.866 | 0.898 | 0.882 | 0.767 |
| 40 | 0.879 | 0.913 | 0.896 | 0.795 |
| 50 | 0.877 | 0.918 | 0.897 | 0.800 |
| 60 | 0.877 | 0.918 | 0.897 | 0.799 |
| 70 | 0.879 | 0.920 | 0.899 | 0.804 |
| 80 | 0.881 | 0.920 | 0.900 | 0.806 |
| 90 | 0.874 | 0.920 | 0.897 | 0.797 |
| 100 | 0.872 | 0.922 | 0.897 | 0.795 |
| 110 | 0.870 | 0.918 | 0.894 | 0.791 |
| 120 | 0.874 | 0.922 | 0.898 | 0.797 |
| 130 | 0.885 | 0.922 | 0.904 | 0.808 |
| 140 | 0.883 | 0.924 | 0.904 | 0.808 |
| 150 | **0.885** | 0.926 | **0.906** | **0.812** |
| 160 | 0.885 | 0.924 | 0.905 | 0.810 |
| 170 | 0.883 | 0.926 | 0.905 | 0.810 |
| 180 | 0.879 | 0.926 | 0.903 | 0.808 |
| 190 | 0.874 | 0.929 | 0.902 | 0.806 |
| 200 | 0.872 | 0.929 | 0.900 | 0.804 |
| 210 | 0.872 | 0.924 | 0.898 | 0.798 |
| 220 | 0.872 | 0.926 | 0.899 | 0.800 |
| 230 | 0.874 | 0.926 | 0.900 | 0.802 |
| 240 | 0.870 | 0.926 | 0.898 | 0.798 |
| 250 | 0.877 | 0.924 | 0.900 | 0.804 |
| 260 | 0.877 | 0.924 | 0.900 | 0.804 |
| 270 | 0.872 | 0.924 | 0.898 | 0.798 |
| 280 | 0.872 | 0.924 | 0.898 | 0.798 |
| 290 | 0.877 | 0.924 | 0.900 | 0.804 |
| 300 | 0.872 | 0.922 | 0.897 | 0.795 |
| 310 | 0.870 | 0.924 | 0.897 | 0.796 |
| 320 | 0.874 | 0.922 | 0.898 | 0.797 |
| 330 | 0.870 | 0.920 | 0.895 | 0.793 |
| 340 | 0.870 | 0.920 | 0.895 | 0.793 |
| 350 | 0.868 | 0.920 | 0.894 | 0.793 |
| 360 | 0.870 | 0.918 | 0.894 | 0.791 |
| 370 | 0.870 | 0.918 | 0.894 | 0.791 |
| 380 | 0.868 | 0.916 | 0.892 | 0.789 |
| 390 | 0.870 | 0.916 | 0.893 | 0.789 |
| 400 | 0.870 | 0.916 | 0.893 | 0.789 |
| 410 | 0.872 | 0.916 | 0.894 | 0.791 |
| 420 | 0.872 | 0.916 | 0.894 | 0.791 |
| 430 | 0.872 | 0.916 | 0.894 | 0.791 |
| 440 | 0.872 | 0.916 | 0.894 | 0.791 |
| 450 | 0.872 | 0.916 | 0.894 | 0.791 |
| 460 | 0.872 | 0.916 | 0.894 | 0.791 |
| 470 | 0.872 | 0.916 | 0.894 | 0.791 |
| 480 | 0.872 | 0.918 | 0.895 | 0.793 |
| 490 | 0.874 | 0.916 | 0.895 | 0.793 |
| 500 | 0.877 | 0.916 | 0.896 | 0.797 |
